# Supplementary material for: Process Characterization of Polyvinyl Acetate Emulsions Applying Inline Photon Density Wave Spectroscopy at High Solid Contents
Source: Polymers (Basel). 2021 Feb 23;13(4):669. doi: 10.3390/polym13040669 (PMC7926705; doi:10.3390/polym13040669)
Supplement: Supplementary file 1 [file polymers-13-00669-s001.pdf]

polymers

Article

Process Characterization of Polyvinyl Acetate Emulsions Applying Inline Photon Density Wave Spectroscopy at High Solid Contents

Stephanie Schlappa [1,*], Lee Josephine Brenker [1], Lena Bressel [1], Roland Hass [1,2] and Marvin Münzberg [1]

[1] Department of Physical Chemistry, innoFSPEC, University of Potsdam, Am Muehlenberg 3, 14476 Potsdam, Germany; josie.brenker@googlemail.com (L.J.B.); bressel@uni-potsdam.de (L.B.); rh@pdw-analytics.de (R.H.); marvin.muenzberg@uni-potsdam.de (M.M.)
[2] PDW Analytics GmbH, Geiselbergstraße 4, 14476 Potsdam, Germany; rh@pdw-analytics.de
* Correspondence: stephanie.schlappa@uni-potsdam.de; Tel.: 0049 331 977 206225

Citation: Schlappa, S.; Brenker, L.J.; Bressel, L.; Hass, R.; Münzberg, M. Process Characterization of Polyvinyl Acetate Emulsions Applying Inline Photon Density Wave Spectroscopy at High Solid Contents. *Polymers* **2021**, *13*, 669. https://doi.org/10.3390/polym13040669

Academic Editor: Maria Paulis
Received: 30 January 2021
Accepted: 19 February 2021
Published: 23 February 2021

Publisher's Note: MDPI stays neutral with regard to jurisdictional claims in published maps and institutional affiliations.

Copyright: © 2021 by the authors. Licensee MDPI, Basel, Switzerland. This article is an open access article distributed under the terms and conditions of the Creative Commons Attribution (CC BY) license (http://creativecommons.org/licenses/by/4.0/).

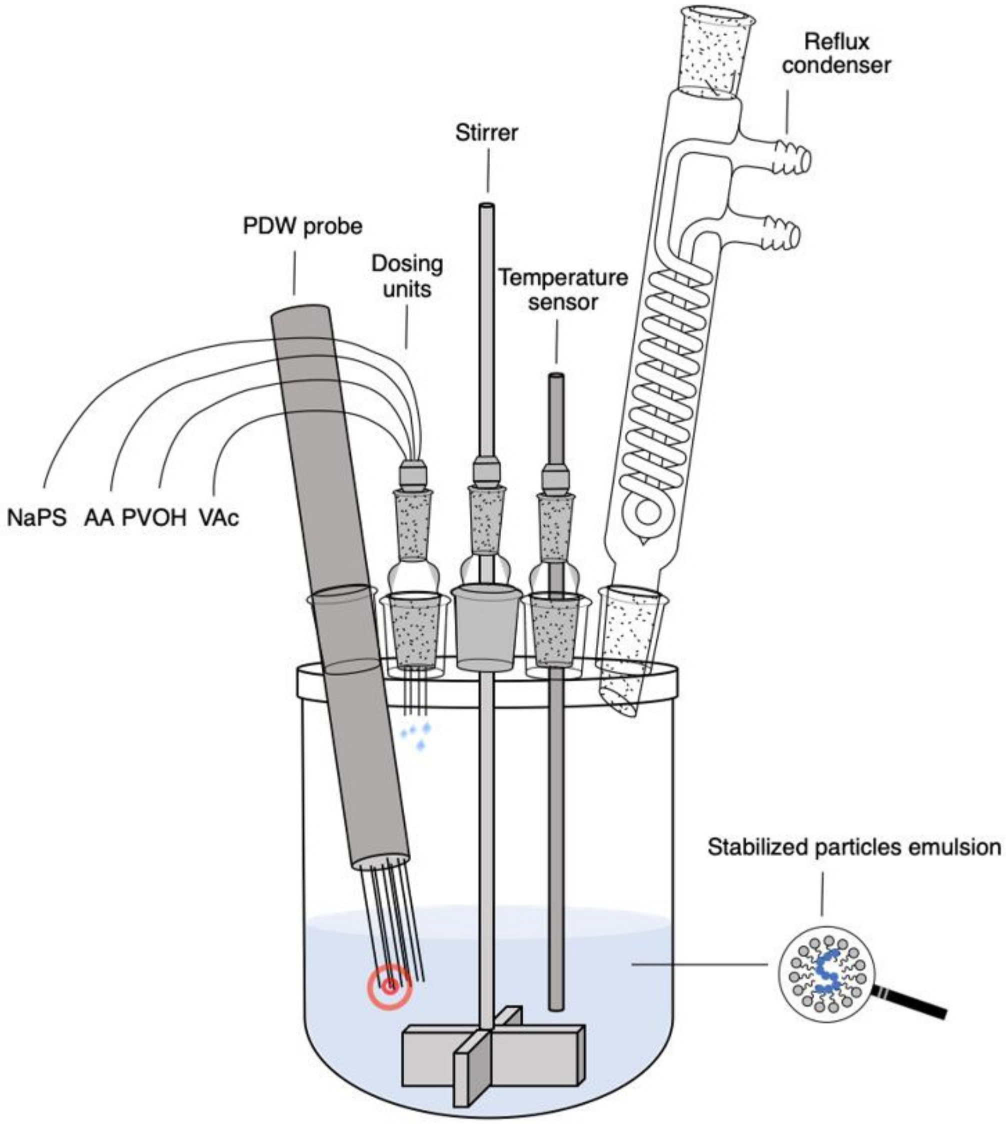

Figure S1. Schematic description of the reactor Set-Up. 1 L automated Lab reactor equipped with automated temperature control, stirring control, dosing control, overhead reflux condenser, and N_2 purging. The PDW inline probe is directly inserted into the reactor via an inlet port.

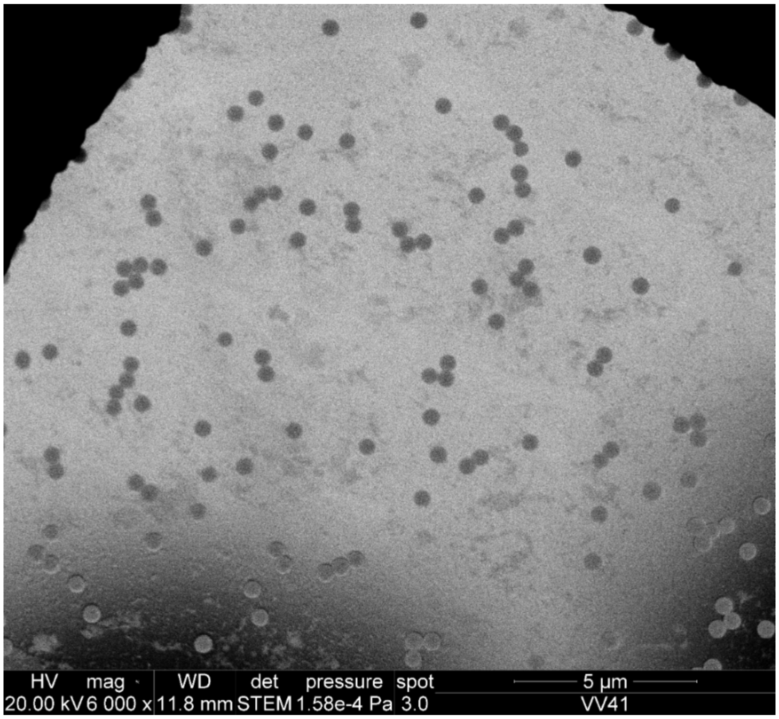

Figure S2. Electron microscopy picture of particles produced during synthesis VAc_1.5.

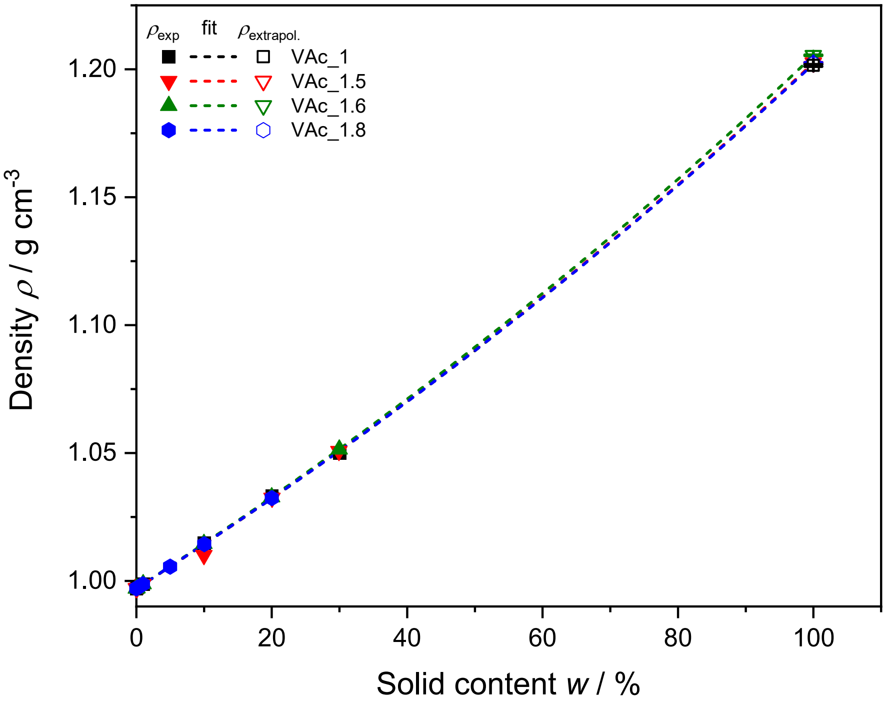

Figure S1 Density of the dispersions of VAc_1, VAc_1.5, VAc_1.6, and VAc_1.8 measured with a densitometer at different concentrations (solid symbols) as well as extrapolated density of the particles (open symbols).

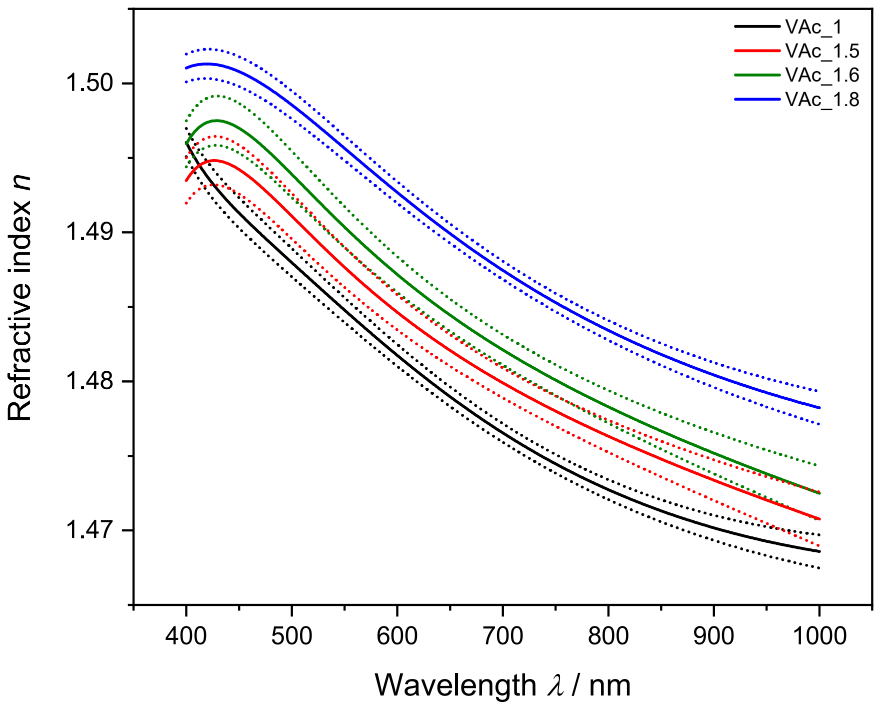

Figure S4. Refractive index of the particles from syntheses VAc_1, VAc_1.5, VAc_1.6, and VAc_1.8 extrapolated from values of a concentration series of the dispersions measured with a refractometer at seven wavelengths and inter- and extrapolated to wavelengths between 400 nm and 1000 nm. Confidence intervals with +95% and –95% are shown as dashed lines.
